# Analysis of Secreted Proteins and Potential Virulence *via* the ICEs-Mediated Pathway of the Foodborne Pathogen *Vibrio parahaemolyticus*

**DOI:** 10.3389/fmicb.2021.612166

**Published:** 2021-03-02

**Authors:** Yu He, Shuai Wang, Kaiwen Wang, Jinwei Zhou, Zhi Han, Fengjiao Sun

**Affiliations:** ^1^College of Food (Biotechnology) Engineering, Xuzhou University of Technology, Xuzhou, China; ^2^Key Construction Laboratory of Food Resources Development and the Quality Safety in Jiangsu, Xuzhou University of Technology, Xuzhou, China; ^3^School of Energy and Power Engineering, Jiangsu University, Zhenjiang, China; ^4^Logistics & Security Department, Shanghai Civil Aviation College, Shanghai, China

**Keywords:** *Vibrio parahaemolyticus*, bacterial secretion systems, secreted protein, integrative and conjugative elements, dihydrolipoamide dehydrogenase

## Abstract

*Vibrio parahaemolyticus* uses bacterial secretion systems and integrative and conjugative elements (ICEs) to induce various diseases and to adapt to harsh environments, respectively. Information pertaining to the identity of secreted proteins and functional characterization of ICEs has been previously reported, but the relationship between these elements remains unclear. Herein we investigated secreted proteins of *V. parahaemolyticus* strains JHY20 and JHY20△ICE using two-dimensional gel electrophoresis and LC-MS/MS, which led to the identification of an ICE-associated secreted protein – dihydrolipoamide dehydrogenase (DLDH). Considering the data related to its physical and biochemical characterization, we predicted that DLDH is a novel immunogenic protein and associated with virulence in JHY20. Our findings indicate a potential relationship between ICE-associated transport and secreted proteins and shed light on the function of such transport mechanisms. We believe that our data should enhance our understanding of mobile genetic elements.

## Introduction

*Vibrio parahaemolyticus* is an important foodborne pathogen and is widely found in seafood such as abalone, shrimp, and crab. In addition, it has been detected in freshwater and marine fish, pickles, livestock and poultry meat, eggs, and other foods (Darling et al., 2004; Sangadkit et al., 2020). In 1950, an outbreak of food poisoning caused by sardines occurred in Osaka, Japan, causing 272 people to contract diarrhea and leading to 20 deaths (Fujino et al., 1965). This was the first report of a food poisoning incident caused by *V. parahaemolyticus* in the world. Since then, food poisoning incidents caused by this bacterium have been often reported. *Vibrio parahaemolyticus* is widespread in the environment, but only a few strains are pathogenic; its pathogenic ability usually depends on its interaction with host cells. *Vibrio parahaemolyticus* produces toxins that directly act on the surface of host cells or enter them, causing cell damage and disrupting normal metabolism or function (Chen et al., 2018). Considering developments within aquaculture and misuse of antibiotics, bacterial strains are encountering strong environmental selection pressure, which has led to the evolvement of various antienvironmental stress mechanisms, consequently making clinical treatment challenging.

Some strains of *V. parahaemolyticus* evidently harbor SXT-R391 integrative and conjugative elements (ICEs), which serve as the main carrier in the horizontal transfer of bacterial resistance genes and other adaptive functional genes (Saharuetai et al., 2020). They can carry a large quantity of genetic information, such as antibiotic resistance gene clusters, heavy metal resistance genes, hydrocarbon degradation genes, and virulence islands, and under the action of integrase, *via* horizontal gene exchange, they endow the host with resistance to allow them to adapt to a particular environment. Reverse genes and mediate the effective spread of drug resistance genes among different bacterial groups so that organisms can adapt to external environmental stresses (Michael et al., 2017; Delahay et al., 2018; Tundis et al., 2019; Zihao et al., 2019). In Gram-negative bacteria-harboring ICEs, there are coding genes that are highly similar to the type IV secretion system. During the conjugative transfer, single-stranded DNA is formed by this system from the donor through the fimbriae connecting channel and transferred to the recipient cell (Xiaobin et al., 2018). The bacterial secretion system mediates the transport of macromolecules across the cell membrane, secretes toxic proteins or effectors, and can directly act on the host target site, triggering various stress responses in the host (Mattingly et al., 2018; He et al., 2020; Pontes et al., 2020). Wozniak and Waldor (2010) suggested that ICEs can enhance the toxicity of strains; however, such speculations have not been validated as yet. In addition, novel ICEs of the SXT/R391 family from some of the major bacterial fish pathogens in marine aquaculture, the variable gene content of ICEs of the SXT/R391 family encodes fitness functions beyond those related to antimicrobial resistance and motility regulation and the host range of these elements in the marine environment might be broader than expected (Balado et al., 2013). In recent years, although studies pertaining to the distribution, evolutionary analysis, and transmission mechanisms of ICEs have been conducted in environmental bacteria, little remains known regarding the virulence mechanism of ICEs, which has largely restricted in-depth research on this topic and on pertinent regulatory mechanisms. It is thus pivotal to conduct studies to explore such mechanisms.

In this study, we used the ICE-carrying strain *V. parahaemolyticus* JHY20 (*tdh^−^, trh^−^*), which has a clear genetic background, and an ICE-deletion mutant strain and a “back-mutant” strain were constructed for the first time. Using comparative secretomics, bioinformatics, and other methods, we comprehensively analyzed secreted proteins, the release of which is mediated by ICEs, and also studied their effects on stress resistance and virulence, with the aim of identifying the key nodes of ICEs that regulate virulence and enhance environmental adaptability. Our results shed light on the mechanisms underlying virulence in *V. parahaemolyticus* and provide a theoretical basis to support the control the spread of this pathogen.

## Materials and Methods

### Construction of a Mutant Strain

JHY20 used in this study was recently isolated and identified by (He et al. 2019). As previously reported (Sun, 2015), we established a highly effective method to screen multiple-drug resistant (MDR) bacteria carrying the ICEs. Antimicrobial activity and heavy metal susceptibility of the *V. parahaemolyticus* strains were examined using standard Kirby-Bauer disk diffusion method and dilution susceptibility tests according to the Clinical and Laboratory Standards Institute (CLSI, United States, 2012 Edition), all showing distinct resistance to 10 antimicrobial agents belonging to six drug classes tested. In addition, a wide heavy metal resistance profile was also detected, displaying strong resistance to Cd, Cu, Zn, and Hg. The conjugation experiments demonstrated the capacities of active self-transmission of the SXT/R391-like ICEs between *V. parahaemolyticus* and *Escherichia coli* MG1655 strains. Eight Proteus vulgaris strains harboring SXT/R391 family of ICEs, isolated from aquatic products in Xuzhou, China, were obtained using the new method. ICEs in JHY20 were eliminated using sodium dodecyl sulfonate at a final concentration of 0.2% (w/v) in tryptic soy broth, combined with a sublethal growing temperature of 42°C. A mutant strain of JHY20 with ICE deletion (JHY20△ICE) was eventually obtained; the elimination rate of typical antibiotic resistance was 100%.

### Bacterial Strain and Culture Conditions

Bacterial strains were usually grown in Luria-Bertani (LB) broth (Land Bridge, Beijing, China) supplemented with 3% NaCl (final concentration, pH 8.5) at 37°C with shaking. To collect secreted proteins, JHY20 and JHY20△ICE were aerobically cultured in LB medium at 37°C without shaking.

### Collection of Extracellular Products (ECP)

Extracellular products (ECP) were collected using a method previously described by Lee et al. (1997) and He et al. (2020), with slight modifications. Two bacterial swabs were suspended in 5ml LB broth supplemented with 3% NaCl (final concentration, pH 8.5) and spread onto cellophane overlying LB agar (Land Bridge) containing 3% NaCl (final concentration, pH 8.5), followed by incubation at 37°C for 12h. Then, 5ml PBS was added to the surface of the cellophane, stirred, and this bacterial suspension was centrifuged at 12,000rpm for 15min at 4°C. The obtained pellet was discarded, and the supernatant was passed through a membrane filter (pore size, 0.22μm; Millipore, Bedford, United States) and then stored at −20°C.

### Sodium Dodecyl Sulfate-Polyacrylamide Gel Electrophoresis (SDS-PAGE)

The protein samples obtained from JHY20 and JHY20△ICE were analyzed by sodium dodecyl sulfate-polyacrylamide gel electrophoresis (SDS-PAGE), as previously reported by Li et al. (2013), with slight modifications. Before SDS-PAGE, protein content was quantified to 6mg/ml using the BCA protein assay kit (Sangon, Shanghai, China), according to manufacturer instructions. Each sample (12μl) was mixed with 4× SDS-PAGE loading buffer (4μl; Japan TaKaRa BIO, Dalian Company, Dalian, China) containing 4% SDS, 200mM dithiothreitol, 40% glycerol, 40mM Tris-HCl (pH 8.0), and 0.032% bromophenol blue, and then heated in boiling water for 10min. The samples (15μl) were loaded onto a 5% (w/v) and 12% (w/v) stacking and resolving gels, respectively, which were hand-casted using a Mini-Protean III system (Bio-Rad Laboratories, Hercules, California, United States). The gels were run at a constant voltage of 60V for 30min; the voltage was then increased to 120V, and the gels were run for 1.5h. Each gel was stained in a solution containing 0.25% (w/v) Coomassie Blue R-250, 5% (v/v) ethanol, and 7.5% (v/v) acetic acid, and then distained in a solution containing 7.5% (v/v) acetic acid and 25% (v/v) ethanol.

### Secreted Protein Collection

To collect secreted proteins, JHY20 and JHY20△ICE were cultured in LB broth at 37°C without shaking for 5h. Bacterial cultures (1L) were centrifuged at 12,000rpm for 5min at 4°C; the supernatant thus obtained was passed through a membrane filter (pore size, 0.22μm; Millipore) was used to extract total protein, and the pellet was used to extract total RNA. Total protein content was measured using the BCA protein assay kit (Sangon) with bovine serum albumin as the standard; the protein was quantified to 1μg/μl, and the samples were stored in 1ml aliquots. The samples were precipitated by adding trichloroacetic acid to a final concentration of 10% (v/v) and incubated on ice overnight. Next day, proteins were collected by centrifugation at 12,000rpm for 30min at 4°C. Ice-cold acetone (50ml) was used to wash the obtained pellets; this step was repeated five times. The pellet was then dried and stored at −80°C.

### Two-Dimensional Gel Electrophoresis (2D-GE)

The secreted protein samples were dissolved in 1ml of rehydration solution, which contained 8M urea, 4% (w/v) 3-[(3-cholamidopropyl)-dimethylammonio]-1-propanesulfonate, 2mM tributyl phosphine (Bio-Rad Laboratories), 0.2% (v/v) Bio-Lyte 3/10 ampholyte (Bio-Rad Laboratories), and 0.0002% (w/v) bromophenol blue; bromophenol blue was added to the samples just before performing two-dimensional gel electrophoresis (2D-GE). The assays were performed in triplicate. The gels were analyzed and spot detection and quantification were performed using PDQuest Advanced-8.0.1 (Bio-Rad Laboratories), resulting in standardized synthetic images.

Isoelectric focusing was performed as the first dimension using an immobilized pH gradient (IPG) strip (pH 3–10/NL, 17cm; Bio-Rad Laboratories). The protein samples (300μl) were dissolved in the rehydration solution, applied to the strips, and rehydration was allowed to proceed for 17h at 17°C. The samples were then focused using an eight-step program (50V for 1h with slow ramping; 100V for 2h with slow ramping; 500V for 1h with slow ramping; 1,000V for 1h with slow ramping; 2,000V for 1h with linear ramping; 4,000V for 1h with linear ramping; 6,000V for 3h with rapid ramping; and 10,000V with rapid ramping until 70,000V was reached). Upon completion of electrophoresis in the first dimension, the IPG strip was incubated in equilibration buffer I (6M urea, 0.05M Tris, 2% SDS, and 20% glycerol) containing 2% (w/v) dithiothreitol for 15min before being washed for a further 15min in equilibration buffer II (6M urea, 0.05M Tris, 2% SDS, and 20% glycerol) containing 2.5% (w/v) iodoacetamide (Sigma).

Separation in the second dimension was performed using a 15% SDS-polyacrylamide gel. After electrophoresis, the gels were stained using the Silver Stain™ Plus kit (Bio-Rad Laboratories), as per manufacturer instructions. Before analysis, the stained gels were stored in a solution containing 1% acetic acid at 4°C, and then digitized with a UMAX PowerLook 2100XL-USB scanner (Veutron Corporation).

### LC-MS/MS

Visible discriminative spots present on the stained gels were excised and subjected to digestion by adding freshly prepared sequencing grade modified trypsin (1:50; Promega, Madison, WI, United States), followed by overnight incubation at 37°C. Subsequently, peptides were using LC-MS/MS with a serially coupled microcolumn in a paradigm HPLC system (Shimadzu Inc., Kyoto, Japan) coupled with an microTOF-QΙΙ mass spectrometer (Bruker, Billerica, MA, Germany). Twenty-five micrograms of secreted protein digests were loaded onto a C18 trap column (300μm i.d. × 5mm, 5μm; Thermo Scientific, San Jose, CA, United States) for desalting, followed by separation on a C18 trap column self-packed with Venusil × BPC (0.3mm i.d., Agela Technologies). The binary mobile phase consisted of solvents A (0.1% HCOOH + H_2_O, v/v) and B (0.1% HCOOH + ACN, v/v), and the flow rate was 400nl/min. Peptide separation was performed with a mobile-phase gradient, as follows: 0–4min, 5% B; 4–30min, 5–40% B; 30–35min, 40–80% B; and 35–45min, 80% B. For ESI-MS/MS, the spray voltage was set at 1.5kV, and the collision energy was set to a value that depended on the *m/z* of the peptide. The temperature of the ion-transfer capillary was set at 150°C, and the scanning range of relative molecular mass was 50–2,200. The scan time was 30min. System control and data collection were achieved using the Bruker Data Analysis 4.0 software.

### Data Analyses

Data analysis was performed based on cumulative total proteins identified in three replicative runs. The collected MS data files were converted to the MASCOT generic format, and the combined files of the three replicative runs were sent to a MASCOT server (v2.3.01, Matrix Science) for automated peptide identification using the UniProt database [Bacteria (Eubacteria) 20140225]. The following MASCOT settings were used: carbamidomethyl was specified as a fixed modification and methionine oxidation was specified as variable modifications. Two missed cleavages were allowed. The precursor mass tolerance was set to 0.1Da and the fragment mass tolerance to 0.1Da. The significance threshold was *p* < 0.05. The ion score cut-off depended on the sample.

### RNA Extraction

Total RNA extraction from JHY20 and JHY20△ICE (cultured for 5h) was performed using the RNeasy Protect Bacteria Mini Kit (QIAGEN Biotech Co. Ltd., Hilden, Germany). The extracted RNA was purified with the RNeasy Mini Kit (QIAGEN), according to manufacturer instructions, and treated with RNase-free DNase (QIAGEN Biotech Co. Ltd., Hilden, Germany). RNA quality was monitored by agarose gel electrophoresis and quantity was determined using a Synergy 2 Multi-Mode Microplate Reader (BioTek Instruments, Inc., Vermont, United States).

### Quantitative Real-Time Reverse Transcription-Polymerase Chain Reaction (RT-qPCR)

*dldh* [encoding dihydrolipoamide dehydrogenase (DLDH)] expression in JHY20 and JHY20△ICE was determined by Quantitative real-time reverse transcription-polymerase chain reaction (RT-qPCR). Primers (Table 1) were designed using the Primer 5 software, and 16S rRNA was used as the reference gene, as previously described by Chen et al. (2009). The reverse transcription reaction was performed using the PrimeScript® RT Reagent kit with gDNA Eraser (Perfect Real Time; Japan TaKaRa BIO, Dalian Company, Dalian, China), as per manufacturer instructions. The reaction volume (20μl) contained 10μl FastStart Universal SYBR Green Master (ROX), 5μM of each of the oligonucleotide primers, 2μl template cDNA, and appropriate volume of sterile ddH_2_O (Roche, Basel, Switzerland). A negative control without any cDNA was included in each assay. Melting curve analysis of amplification products was performed at the end of each PCR to confirm that only one product was amplified and detected. RT-qPCR was performed using a 7500 Fast Real-Time PCR System (Applied Biosystems, Foster City, CA, United States) with the following conditions: initial denaturation at 50°C for 2min, followed by 40cycles of denaturation at 95°C for 15s and primer annealing at 60°C for 60s.

**Table 1:** Primers used for real-time quantitative PCR.

Gene	Designation	Primer sequence (5'→3')	Product size (bp)
16s rRNA	16s497F	GAAGAAGCACCGGCTAACTCC	101
	16s597R	AACAAACCACCTGCATGCG	
*dldh*	dldhF	GACTCTACTGACGCTCTTG	201
	dldhR	GAACTTGTCTTTGATACGC	

## Results

### Analyses of ECP

**Figure 1:**
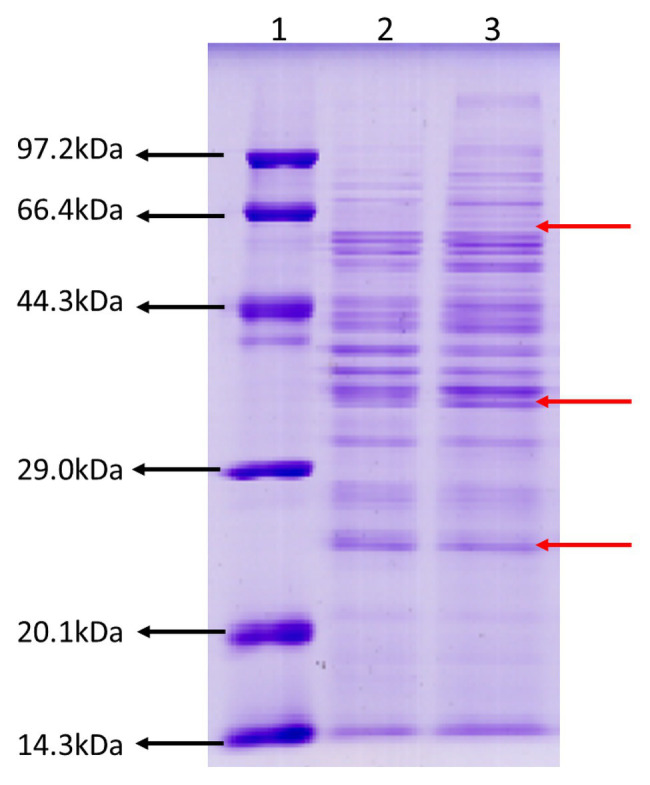
SDS-PAGE analysis of the *Vibrio parahaemolyticus* extracellular products. Protein molecular weight marker (Tiangen, Cat. No. DPP530S) **(Lane 1)**, an extracellular product of *Vibrio parahaemolyticus* JHY20△ICE **(Lane 2)**, and *Vibrio parahaemolyticus* JHY20 **(Lane 3)** cultured under the same condition at 37°C for 12h.

To gain an insight into ECP secreted by JHY20 and JHY20△ICE, we analyzed extracellular proteins using SDS-PAGE. The protein profiles appeared similar (Figure 1), and the majority of extracellular proteins secreted by the strains ranged from 25 to 66kDa in size. Distinct protein bands were observed to be located at 25, 40, and 60kDa, and these were thus further investigated using 2D-GE.

### Comparative Analysis of Secreted Proteins by 2D-GE

**Figure 2:**
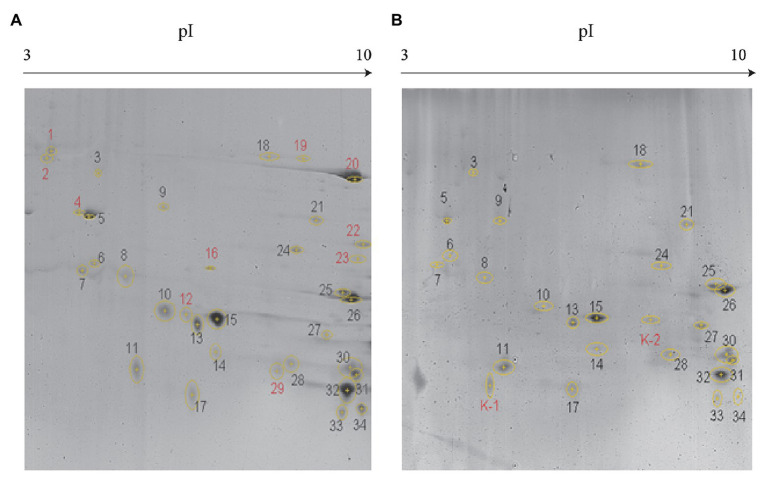
Identification of secreted protein by two-dimensional gel electrophoresis (2-DE). Discrimination of secreted proteins between JHY20 **(A)** and JHY20△ICE **(B)**. Twelve discrepancy protein spots were detected in the supernatant of strain, indicated by numbers in red.

As evident from Figure 2, secreted proteins of both the strains were concentrated at the center of the IPG strip. In total, 34 and 26 protein spots were derived from JHY20 and JHY20△ICE, respectively. With the consensus summary, 24 spots were found to match across the two strains. Ten and two spots were exclusively detected in JHY20 and JHY20△ICE, respectively. In case of the mutant strain, two of the discriminative spots, which was located near the acidic and high molecular weight region, was further analyzed by LC-MS/MS. Table 2 presents data pertaining to spot comparison and matching.

**Table 2:** Spots comparison and matching summary.

Strain	Spots	Matched spots	Unmatched spots	Unmatched spots No.
JHY20	34	24	10	1, 2, 4, 12, 16, 19, 20, 22, 23, 29
JHY20△ICEs	26	24	2	K-1, K-2

### Identification of Secreted Proteins by LC-MS/MS

**Figure 3:**
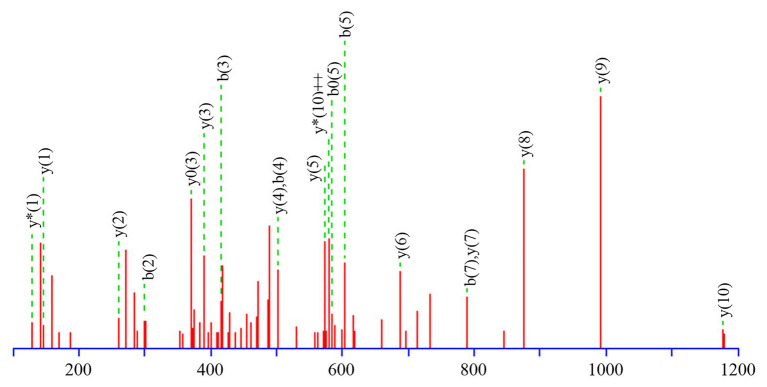
b and y ions annotation of MS/MS fragmentation of IWDSTDALELK.

**Figure 4:**
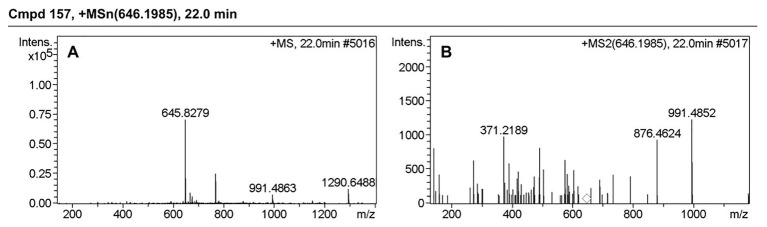
MS and MS/MS spectrum of IWDSTDALELK. **(A)** MS spectrum of IWDSTDALELK. **(B)** MS/MS spectrum of IWDSTDALELK.

Table 3 presents information on secreted proteins that were analyzed by LC-MS/MS. The results were in agreement with those of previous studies based on SDS-PAGE and 2D-GE; most differentially expressed proteins have been correspondingly analyzed in previous studies (He et al., 2015, 2020). In addition, we identified one ICE-associated secreted protein – DLDH. The results revealed 100% identity at the amino acid level to *vp2517*, which encodes DLDH in *V. parahaemolyticus* RIMD2210633. The MS and MS/MS spectra for DLDH (molecular mass, 51.241kDa; pI, 5.72) are shown in Figures 3, 4, respectively. We found the parent ion *m/z* was 645.8279 in the MS spectrum, and annotate b and y ions to the MS/MS Fragmentation of IWDSTDALELK.

**Table 3:** Identification of the protein spots on the secretome profiles by LC-MS/MS analysis.

Protein spot No.	Uniprot No.	Protein	Gene (RIMD2210633)	MW (Da)	pI	Score	Sequence coverage	Putative function
1	O50286	Dihydrolipoyl dehydrogenase	*vp2517*	51,241	5.72	2,971	76%	The branched-chain alpha-keto dehydrogenase complex catalyzes the overall conversion of alpha-keto acids to acyl-CoA and CO_2_.
2	Q87PK2	Hypothetical protein	*vp1501*	32,363	5.38	4,485	72%	–
4	Q8GRF5	Ribosome-recycling factor	*vp2315*	20,467	6.03	657	77%	Responsible for the release of ribosomes from messenger RNA at the termination of protein biosynthesis. May increase the efficiency of translation by recycling ribosomes from one round of translation to another.
12	C3LMW6	50S ribosomal protein L25	*vp1210*	10,483	6.82	568	37%	Binds to the 5S RNA in the ribosome where it forms part of the central protuberance.
16	–	–	–	–	–	–	–	–
19	–	–	–	–	–	–	–	–
20	A0KQA8	50S ribosomal protein L1	*rplA*	24,585	9.61	1,490	65%	Binds directly to 23S rRNA. The L1 stalk is quite mobile in the ribosome, and is involved in E site tRNA release. UniRule annotation. Protein L1 is also a translational repressor protein, it controls the translation of the L11 operon by binding to its mRNA.
22	Q87SZ8	50S ribosomal protein L6	*rplF*	18,905	9.71	87	52%	This protein binds to the 23S rRNA, and is important in its secondary structure. It is located near the subunit interface in the base of the L7/L12 stalk, and near the tRNA binding site of the peptidyltransferase center.
23	Q87SI5	50S ribosomal protein L13	*rplM*	15,951	9.64	304	50%	This protein is one of the early assembly proteins of the 50S ribosomal subunit, although it is not seen to bind rRNA by itself. It is important during the early stages of 50S assembly.
29	Q9KQS9	DNA-binding protein HU-beta	*hupB*	9,417	9.58	154	44%	Histone-like DNA-binding protein which is capable of wrapping DNA to stabilize it, and thus to prevent its denaturation under extreme environmental conditions.
K-1	–	–	–	–	–	–	–	–
K-2	–	–	–	–	–	–	–	–

–*: Indicate no identification result*.

**Figure 5:**
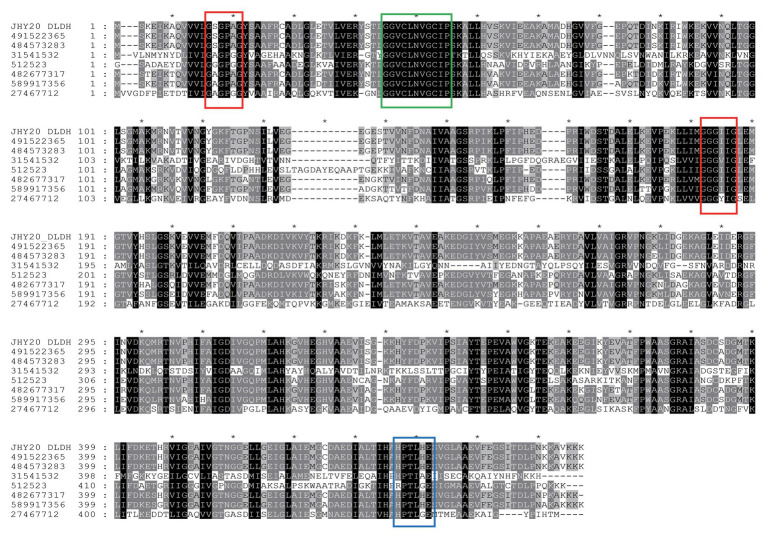
A multiple sequence alignment of *Vibrio parahaemolyticus* strain JHY20 dihydrolipoamide dehydrogenase (DLDH) and a selected set of bacterial DLDH proteins. Numbers above the alignments indicate relative positions of the entirely aligned sequences. Identical amino acid residues, as well as the conserved ones (>50% of the sequences), are highlighted in black and in gray, respectively. The FAD and pyridine nucleotide-binding motifs (GXGXXG) are in red box. Two catalytic cysteine residues (GGXCXXXGCXP) and His-Glu pair residues (HXXXXE) are indicated by a green box and blue box, respectively. The DLDH sequences chosen for the analysis are obtained from GenBank and NCBI databases: GI: 491522365: *Vibrio harveyi*; GI: 484573283: *Vibrio alginolyticus*; GI: 31541532: *Mycoplasma gallisepticum* str. R(low); GI: 512523: *N. meningitides* serogroup B; GI: 482677317: *Escherichia coli*; GI: 589917356: *Aeromonas caviae*; GI: 27467712: *Staphylococcus epidermidis* ATCC12228. JHY20_DLDH, obtained in this study.

**Figure 6:**
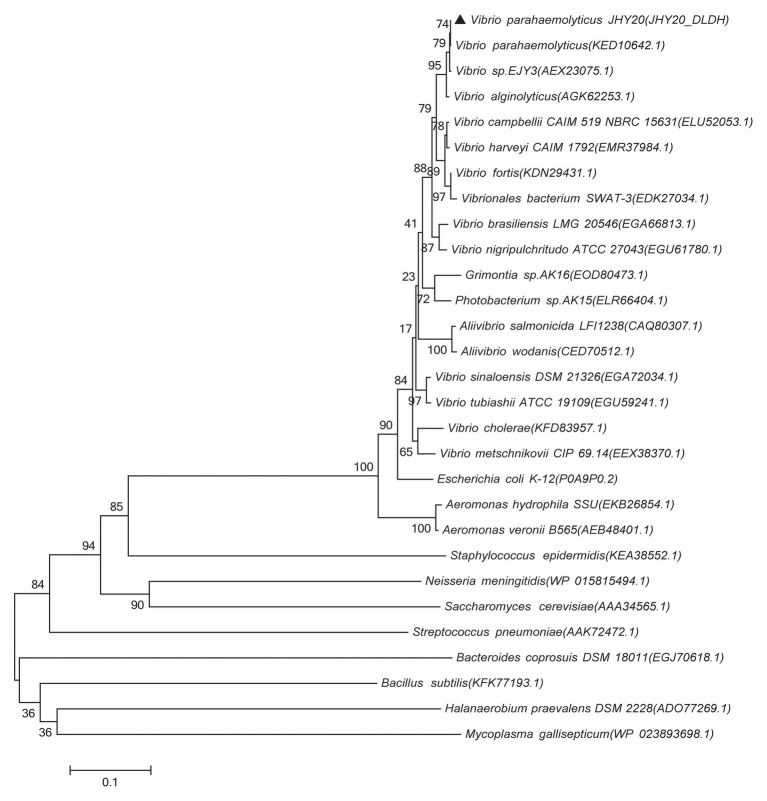
Phylogenetic tree showing evolutionary relationship between the *Vibrio* sp. DLDH and some characterized bacterial DLDH and related proteins. Based on the DLDH amino acid sequences derived from JHY20 in this study and from some known DLDH in the public databases, the neighbor-joining phylogenetic tree was constructed by using the MEGA 6.06. Bootstrap percentages are shown at nodes. The scale bar represents 0.1 changes per amino acid. Filled cycles denote the bacterial DLDH proteins that have been characterized for their activity or function, whereas JHY20 DLDH identified in this study is marked with a solid triangle.

Based on DLDH sequences obtained in this study and a selected set of DLDH homologs identified in public databases, we selected bacterial DLDH proteins that have been characterized for their activity or function; multiple sequence alignments (Figure 5) were then performed and phylogenetic trees were constructed (MEGA v6.06) to gain an insight into their diversity and classification (Figure 6).

The DLDH protein of JHY20 was noted to be 475 amino acids in length and was highly identical (40–99%) at the amino acid level to other characterized enzymes: *Vibrio harveyi* (GI: 491522365, 99%), *Vibrio alginolyticus* (GI: 484573283, 99%), *Mycoplasma gallisepticum* (GI: 31541532, 97%), *Neisseria meningitidis* (GI: 512523, 60%), *E. coli* (GI: 482677317, 88%), *Aeromonas caviae* (GI: 589917356, 83%), *Streptococcus pneumoniae* D39 (GI: 17223674, 40%), and *Staphylococcus epidermidis* ATCC 12228 (GI: 27467712, 44%). As evident from Figure 5, DLDH of JHY20 contained four motifs: FAD and pyridine nucleotide-binding motifs (GXGXXG), two catalytic cysteine residues (GGXCXXXGCXP) involved in the redox-active disulfide site, and His-Glu pair residues (HXXXXE). All of them are essential for the activity of flavin-containing pyridine nucleotide-disulfide oxidoreductase family enzymes (Youn et al., 2002; Kurokawa et al., 2010). Using Prosite^1^ for analyzing the common motifs in DLDH, it was predicted that its amino acid sequence had 27 hits: one pyridine nucleotide-disulfide oxidoreductases class-I active site, 11 N-myristoylation sites, one N-glycosylation site, six casein kinase-II phosphorylation sites, five protein kinase C phosphorylation sites, two amidation sites, and one tyrosine kinase phosphorylation site.

As shown in Figure 6, the majority of previously reported DLDH proteins that have been characterized for their activity or function in *Vibrio* sp., *E. coli*, *A. caviae*, and *N. meningitidis* were distributed in the same cluster, whereas in case of *S. epidermidis* and *M. gallisepticum*, they were present in two distinct clusters. These bacterial species thus seem to be distantly related phylogenetically. Further, despite different bacterial origins, the DLDH proteins appear to have a common ancestor in their evolutionary histories.

### Transcription of *dldh*

To confirm our prediction that DLDH is a secreted protein in JHY20 and that it is secreted *via* the ICE-associated apparatus, we quantitated *dldh* levels using RT-qPCR. The average relative quantitative levels of *dldh* in JHY20 and JHY20△ICE were 1.045 and 0.938, respectively. This finding indicated that the *dldh* level did not significantly change at the transcriptional level upon the elimination of ICEs, but the DLDH protein could not be detected in the ECP of the mutant strain. It is preliminarily inferred that ICEs in JHY20 mediate the secretory pathway of DLDH. In addition, further studies are warranted to comprehensively elucidate the function of DLDH.

## Discussion

Dihydrolipoamide dehydrogenase serves as a metabolic factor in bacteria. In *E. coli*, it is an integral component of all three *α*-ketoacid dehydrogenase multienzyme complexes as well as the glycine decarboxylase complex, which is located in the cytoplasm. Both DLDH and its substrate, namely lipoic acid, have been implicated in glycine degradation and a sugar-binding protein-dependent transport system (Richarme, 1985; Freudenberg et al., 1989; McMillan et al., 2005). DLDH was recently reported to also play a key role in pathogenicity. Molecular structure analyses of DLDH from the outer membrane of *N. meningitidis* revealed that the main domain contains an extended loop of 32 residues at the surface of the protein, and this additional segment corresponds to the major antigenic determinant of the protein; this property is currently under evaluation for large scale production (Li de la Sierra et al., 1997). Further, DLDH has been reported to play an important role in pneumococcal infection; DLDH-negative bacteria grew normally *in vitro* but were avirulent in sepsis and lung infection models in mice. Thus, it seems to be necessary for the survival of pneumococci within the host, as described by Smith et al. (2002). In addition, DLDH was recognized by antisera as the bacterial receptors that bind host factors, which is one of the cell wall-associated proteins isolated from *S. epidermidis* after growth in serum (Sellman et al., 2005). In *V. harveyi* and *V. alginolyticus*, Western blot analysis was performed on whole-cell lysates using rabbit antiserum, and DLDH was found to be one of the whole-cell proteins with specific immunoreactivity and identified as a novel immunogenic protein (Pang et al., 2010, 2013). DLDH has also been associated with virulence in *M. gallisepticum* using signature sequence mutagenesis (Hudson et al., 2006). In case of the tellurite-resistant bacterium *A. caviae* ST, DLDH could reduce tellurite to elemental tellurium (Arenas et al., 2014). DLDH were located on the outer membrane of *Vibrio splendidus*, determined to play important roles in adhesion to different matrices and the adhesive ability of *V. splendidus* reduced more than 50% when DLDH was defective (Fa et al., 2019). The other important function of DLDH as a complement regulator binding protein that might play an important role in virulence of *Pseudomonas aeruginosa* (Teresia et al., 2015). Further, DLDH has been reported to be an autoantigen specific to patients with endometrial cancer (Yoneyama et al., 2014). In addition, numerous studies have reported that DLDH is located in the cytoplasm, but very few have reported it to be a secreted protein. Jiang et al. (2006) and Wu et al. (2008) described it to be an extracellular enzyme for the first time in *Bacillus subtilis* WY34, but its function was unknown. Considering these data, we predict that DLDH is a novel immunogenic protein and is associated with the virulence of JHY20.

*Vibrio parahaemolyticus* is a Gram-negative, halophilic bacterium that thrives in warm climates within marine or estuarine environments (Broberg et al., 2011). Virulent strains can cause distinct diseases, including seafood-associated bacterial gastroenteritis, septicemia, and serious wound infections (Boyd et al., 2008). The global dissemination of this pathogen underscores the importance of understanding its many virulence factors and their effects on the human host; the involvement of the bacterial secretion systems has led to the evolvement of numerous *V. parahaemolyticus* species with varying degrees of pathogenicity (Cascales, 2008; Broberg et al., 2011). To allow adaptation to harsh environments, ICEs enable bacteria to become resistant to multiple antibiotics and some complex new traits through horizontal gene transfer (Dobrindt et al., 2004; Balado et al., 2013). They encode a wide variety of genetic information that can be beneficial under certain environmental conditions (Ravatn et al., 1998; Bi et al., 2012; Guerillot et al., 2013). Information pertaining to the functional characterization of ICEs in their hosts is still very limited, and not many studies have explored their effects on secreted proteins. We herein analyzed the ICE-harboring strains of *V. parahaemolyticus* JHY20 isolated from shrimps in Xuzhou, Jiangsu, China (Song et al., 2013). Our findings pertaining to the DLDH protein were consistent with those previously reported in the literature (Park et al., 2004; Ono et al., 2006; Salomon et al., 2013); further, consistent with the findings of Jackman et al. (1990), Moran et al. (2002), McMillan et al. (2005), Jiang et al. (2006), and Wu et al. (2008), we report that DLDH is a secreted protein. We found that it is involved in nine signaling pathways, such as citrate cycle, glycolysis, and gluconeogenesis, and considering the information pertaining to its physical and biochemical characterization, we predicted that DLDH plays a key role in metabolic pathways and cytotoxicity. These results suggest a potential relationship between ICE-associated transport and secreted proteins and shed light on the function of these transport mechanisms.

This study had some limitations. We used 2D-GE to identify secreted proteins; some additional proteins that must be secreted in small quantities or whose pI values were beyond the range of the 2D gels (pI 3–11) used in this study may be present but were not detected. Further, the fact that DLDH was not a part of the ECP of JHY20△ICE raises questions such as how it is transferred by ICE-associated transport and what is its function in the extracellular environment. In addition, many unanswered questions remain to exist: how ICEs mediate virulence in *V. parahaemolyticus*? Do they affect the ability of strains to absorb nutrients in the external environment and promote their resistance to stress? What kind of differential regulation of metabolism is triggered due to the absence of ICEs? The findings from this study should enhance our understanding of mobile genetic elements, and those unresolved questions would facilitate further studies that focus on elucidating the pathogenesis of *V. parahaemolyticus* and detecting diagnostic markers.

## Data Availability Statement

The original contributions presented in the study are included in the article/supplementary material, further inquiries can be directed to the corresponding author.

## Author Contributions

ZH participated in the design and/or discussion of the study. FS established an efficient method for screening the mutant strain. YH, SW, and KW performed all major experiments. SW, YH, and JZ analyzed the data. SW and YH wrote the manuscript. SW and ZH revised the manuscript. All authors contributed to the article and approved the submitted version.

### Conflict of Interest

The authors declare that the research was conducted in the absence of any commercial or financial relationships that could be construed as a potential conflict of interest.

## Footnotes

^1^www.expasy.org/prosite
